# Introgression of *SbERD4* Gene Encodes an Early-Responsive Dehydration-Stress Protein That Confers Tolerance against Different Types of Abiotic Stresses in Transgenic Tobacco

**DOI:** 10.3390/cells11010062

**Published:** 2021-12-27

**Authors:** Rajesh Kumar Jha, Avinash Mishra

**Affiliations:** 1 Division of Applied Phycology and Biotechnology, CSIR–Central Salt and Marine Chemicals Research Institute, G. B. Marg, Bhavnagar 364002, India; rajeshkumarjha2588@gmail.com; 2 Academy of Scientific and Innovative Research (AcSIR), Ghaziabad 201002, India

**Keywords:** abiotic stress, drought, genetic transformation, halophyte, salinity, transgenic

## Abstract

*Salicornia brachiata* is an extreme halophyte that commonly grows on marsh conditions and is also considered a promising resource for drought and salt-responsive genes. To unveil a glimpse of stress endurance by plants, it is of the utmost importance to develop an understanding of stress tolerance mechanisms. ‘Early Responsive to Dehydration’ (ERD) genes are defined as a group of genes involved in stress tolerance and the development of plants. To increase this understanding, parallel to this expedited thought, a novel *SbERD4* gene was cloned from *S. brachiata*, characterized, and functionally validated in the model plant tobacco. The study showed that *SbERD4* is a plasma-membrane bound protein, and its overexpression in tobacco plants improved salinity and osmotic stress tolerance. Transgenic plants showed high relative water, chlorophylls, sugars, starch, polyphenols, proline, free amino acids, and low electrolyte leakage and H_2_O_2_ content compared to control plants (wild type and vector control) under different abiotic stress conditions. Furthermore, the transcript expression of antioxidant enzyme encoding genes *NtCAT*, *NtSOD*, *NtGR*, and *NtAPX* showed higher expression in transgenic compared to wild-type and vector controls under varying stress conditions. Overall, the overexpression of a novel early responsive to dehydration stress protein 4-encoding gene (*SbERD4*) enhanced the tolerance of the plant against multiple abiotic stresses. In conclusion, the overexpression of the *SbERD4* gene mitigates plant physiology by enduring stress tolerance and might be considered as a promising key gene for engineering salinity and drought stress tolerance in crops.

## 1. Introduction

Plants being sessile in nature evolved an adaptive mechanism to perceive, respond, and cope with adverse environmental conditions during their life cycle [1]. Abiotic stresses are major growth-limiting factors that adversely affect the overall plant growth and crop productivity in agriculture. Among the abiotic stresses, dehydration and salinity are the key restrictive factors limiting crop productivity in agriculture through alteration in metabolism and gene expression [2,3]. They also affect the geographical distribution of the plant in nature as well as the growth and development at molecular and physio-biochemical levels that ultimately leads to food security [4]. It occurs more often due to the shortage of irrigation water and rapid global climate change mainly attributed to an increasing world population at an alarming rate [4,5]. In that pretext, improving salinity and drought resistance in crop plants has become an utmost important approach for safeguarding sustainable agriculture and food security [6,7,8].

Similarly, multiple stresses lead to the disruption of various plant physiological responses such as osmotic stress, ionic stress, and the accumulation of excessive reactive oxygen species (ROS) in the plant cell. The primary response of this environmental stress is the induction of the abiotic stress-responsive candidate’s gene that provides tolerance through the production of osmoprotectants and encodes various detoxification proteins, which act to mitigate the deleterious effects of stress signals [9]. These stress signals decipher the up-regulation of gene(s) involved in stress tolerance pathways, which are further classified according to their pattern of gene expression comprising responsive to dehydration (RD), early responsive to dehydration (ERD), cold regulated (COR), cold-inducible (KIN), and low temperature-induced (LTI).

Plants induce a large number of genes under water-deficit conditions, and such genes can be classified according to their response and time of induction, which is further classified as response to dehydration (RD) and early response to dehydration (ERD) [10,11]. Early responsive to dehydration (ERD) genes were categorized into 16 groups based on results demonstrated by southern hybridization that correspond to genes expressed upon dehydration stress for 1 h in *Arabidopsis thaliana* using a differential screening technique [10]. Among the 16 ERD genes, early responsive to dehydration four (ERD4) was identified as a membrane-encoded protein [3].

An extreme halophyte, *Salicornia brachiata* Roxb, is a potential marine agricultural alternative crop and is considered as a reservoir of drought and salt-tolerance genes and promoters [11]. Several promising abiotic stress tolerance genes [12,13] and promoters [14,15,16] from *Salicornia brachiata* have been characterized in the model [17,18] and crop plants [19,20], and researchers have identified novel candidates genes that confer tolerance against abiotic stress by developing transgenic crop plants [21,22]. Nevertheless, the exact function of the gene associated with abiotic stress tolerance is still unknown and needs to be validated through several molecular and physio-biochemical approaches. *SbERD4* overexpressing transgenic tobacco plants resulted in improved tolerance against abiotic stress such as salinity, drought, cold, and heat. To date, it is assumed that the molecular mechanisms associated with plant responses such as change in gene expression, signal transduction pathways, and regulatory networks related to multiple stresses are yet to be ascertained [23].

ERD4 is an integral protein with ten predicated trans-membrane domains reported in Arabidopsis, although its cellular function is yet to be ascertained and needs to be characterized. Nevertheless, it has been identified partially as part of the chloroplast envelops proteome [24,25]. In a previous study, the expressed sequence tag (EST) of *ZmERD4* shows high sequence similarity with both *Oryza sativa* and *A. thaliana* ERD4 genes. Herein, a full-length sequence of the *SbERD4* gene from halophytes *S. brachiata* shows high similarity with the CSC1-LIKE ERD4 sequence of *Beta vulgaris*. In vivo localization analysis confirmed that *SbERD4* was confined in the plasma membrane, which is also supported by in silico analysis. In this study, we present the cloning and functional characterization of an unknown/novel *SbERD4* from *S. brachiata,* which shows improved abiotic stress tolerance in the transgenic tobacco plant. Overexpression of the *ZmERD4* gene from maize confers improved salt and drought tolerance in *A. thaliana* [25]. In this study, transgenic tobacco plants overexpressing the *SbERD4* gene alleviate osmotic and salt stress tolerance by moderating physio-biochemical processes. In agreement with these results, our findings enhance the understanding of an early responsive to dehydration stress protein (*SbERD4*) improving the tolerance of transgenic tobacco to salinity and osmotic stresses and might be considered as a suitable possibility for the development of transgenic crop plants through genetic manipulation.

## 2. Materials and Methods

### 2.1. Cloning of SbERD4 Gene and In Silico Analysis

The *SbERD4* gene was made full length through the rapid amplification of the cDNA end (RACE) technique using an EST (Sal C38; GenBank no. EC906161) clone and gene-specific primers (Table S1). The gene was amplified, cloned, sequenced (from M/s Marogen Inc., Seoul, South Korea), analyzed, and submitted to NCBI as GenBank no. MK629705. The transmembrane domains, conserved domains, and secondary structure of the protein were predicted by ExPasy tools [26].

### 2.2. Subcellular Localization of SbERD4 Gene

To analyze the subcellular localization of the *SbERD4* protein in vivo, the gateway technology was used to construct a translation fusion cassette together with plasmid control RFP (red fluorescence proteins). The translational fusion constructs *SbERD4:RFP* and vector control pSite-4CA-RFP were transformed into an onion epidermal cell using a Biolistic PDS 1000-He particle delivery method (Bio-rad, Hercules, CA, USA). The bombarded onion leaf-peal was kept at 25 °C for 12–18 h in dark, and transient signals were detected under an Axio imager epifluorescence microscope (Carl Zeiss AG, Jena, Germany).

### 2.3. Transcript Profiling of SbERD4 Gene

One-month-old seedlings of *S. brachiata* were transferred to a hydroponic culture medium 1/2 MS with 8/16 dark/light at 25 °C for 14 days for acclimatization. Subsequently, plants were subjected to different abiotic stress treatments such as salinity (500 mM NaCl), osmotic/drought (300 mM mannitol), cold (4 °C), and heat (40 °C) up to 48 h (3, 6, 12, 24, and 48 h). Total RNA was isolated from unstressed and stressed plants using GITC method [27], and cDNA was made using a kit (Superscript II, Invitrogen, Waltham, MA, USA). The quantitative real time (qRT) PCR used for transcript profiling of the *SbERD4* gene using gene-specific RT primers (Table S1), and β-tubulin gene was used as an internal reference for normalizing the expression data. The relative fold expression of the *SbERD4* gene under control and stressed conditions was analyzed by the comparative CT method [28].

### 2.4. Genetic Transformation and Confirmation of Transgenic Plants

The *SbERD4* cDNA was first cloned in a pRT101 vector, subsequently in pCAMBIA1301. The construct *pCAMBIA1301::35SERD4* transformed to tobacco (cv. Petit Havana) using the *Agrobacterium*-mediated leaf disc method [29]. The transformed plants were primarily assessed on hygromycin (50 mgL^−1^). The transgene (*SbERD4*, *hptII*, and *uidA*) was confirmed through PCR amplification (Supplementary Table S1). The expression of reporter gene β-glucuronidase was analyzed through GUS histochemical assay. Potential transgenic lines L2, L13, L14, L15, and L18, showing a high percentage of germination on an antibiotic hygromycin plate, and high histochemical GUS assay were further selected for the onward study.

### 2.5. Seed Germination, Growth Analysis, Leaf Senescence, and Chlorophyll Content Analysis

Seeds of *SbERD4* overexpressing transgenic lines (L2, L13, L14, L15, and L18), vector control (VC), and wild-type (WT) plants were kept on MS media for germination under control (no additional supplementation) and stress conditions (by supplementing 200 mM NaCl for salinity or 300 mM mannitol for osmotic/drought) for 21 days, and the seed-germination percentage was calculated [30,31]. Different growth parameters, including root (RL) and shoot (SL) length, fresh (Fw) and dry (Dw) weight were calculated and compared with control (WT and VC) plants grown under salinity (200 mM NaCl) and osmotic/drought (300 mM mannitol) stress conditions.

Leaf segments of uniform size were cut from transgenic and control (VC and WT) plants and floated on half-MS media (considered as control condition) and media (half-MS) supplemented with salt (200 mM NaCl) and osmotic (300 mM mannitol) stresses for eight days. The phenotyping changes of samples were documented and compared with the control conditions. Furthermore, the total chlorophyll contents of all samples were calculated as per gram fresh weight of tissue [32].

### 2.6. Stress Treatments for the Analysis of Transgenic Plants

Transgenic and control plants were grown and acclimatized in hydroponic 1/2 MS culture medium for 28 days; subsequently, they were exposed to salt (200 mM NaCl), osmotic/drought (300 mM mannitol), cold (4 °C), and heat (40 °C) stress treatments for 24 h. Samples were harvested, stored, and used for the validation of *SbERD4* overexpressing transgenic lines through various physiological and biochemical analyses.

In the second set of controlled field experiments, transgenic and control plants (28 days old) of uniform size were shifted to the soil and subjected with salinity or drought/osmotic stress treatments to analyze their performance under controlled field conditions [33] by irrigating them with NaCl (200 mM) or mannitol (300 mM) solution on alternate days for the next 15 days. Morphological changes of *SbERD4* overexpressing transgenic lines were documented and compared with the control (VC and WT) plants. Subsequently, for stress retrieval study, drought/osmotic stressed plants were re-irrigated with normal water for eight days; consequently, changes in morphology were documented and compared with controls.

### 2.7. Determination of Relative Water Content, Membrane Stability Index, and Electrolyte Leakage

The fresh weight (Fw) of leaf samples of transgenic and control (WT and VC) plants (treated and untreated) was recorded, and samples were incubated in deionized water for 24 h. After incubation, turgid weight (Tw) was measured, which was followed by dry weight (Dw) measurement at 40 °C. The percentage of relative water content (RWC) was calculated [30] by the following equation:

$$RWC\;\left(\%\right)=100\times \left(\frac{Fw-Dw}{Tw-DW}\right).$$

Leaf segments from both transgenic and control (treated and untreated) plants were kept in deionized water. One set was incubated for 40 °C (L1) for 30 min. The second set was incubated at 100 °C (L2) for 10 min. Electrolyte conductivity (EL) was calculated, and the percentage of the membrane stability index (MSI) was determined [34,35,36] as follows:

$$MSI\;\left(\%\right)=100\times \left[1-\left(\frac{L1}{L2}\right)\right].$$

For electrolyte leakage (EL) analysis, first, leaf-surface attached electrolytes were completely removed by rinsing thoroughly with deionized water. Samples were submersed in water (10 mL) for 12 h with a gentle shaking, and electrolyte conductivity (L0) was recorded (Seven Easy conductivity meter, Mettler Toledo, Switzerland). Subsequently, leaf samples were autoclaved (120 °C, 15 min), cooled up to ambient temperature, and electrical conductivity (Lt) was recorded. The percentage of EL (electrolyte leakage) was calculated [34,35,36] using the following equation:

$$EL\;\left(\%\right)=100\times \left(\frac{Lt}{L0}\right).$$

### 2.8. Estimation of Osmoprotectant and H_2_O_2_ Contents

Leaf samples of treated and untreated transgenic and control plants were extracted with aqueous ethanol (70%, *v/v*). Extracts were evaporated for dryness at 50 °C, and resultant residues were dissolved in deionized water. Total and reducing sugars, free amino acids, and polyphenol contents were measured with anthrone–sulfuric acid, DNS, ninhydrin, and Folin–Ciocalteu reagent method, respectively [30,37,38,39]. The proline and H_2_O_2_ contents were quantified using the spectrophotometry method by recording the absorbance at 520 and 560 nm, respectively [40,41].

### 2.9. Ion Content Analysis

Samples were collected from untreated and treated (200 mM NaCl only) plants and dried to a constant weight. A mixture (1:3) of nitric and perchloric acids was used to digest samples by heating up to complete dry. Dried samples were re-dissolved in deionized water, and ion contents (Na^+^ and K^+^) were determined.

### 2.10. Quantitative RT-PCR of ROS Scavenging Genes

Total RNA was isolated using the GITC method [27] from unstressed and stressed transgenic and control plants, and cDNA was synthesized. The expression pattern of antioxidant enzymes encoding genes (*NtSOD*, *NtPOD*, *NtAPX*, and *NtGR*) was studied using quantitative real-time (qRT) PCR, and the β-tubulin gene used as an internal reference for normalizing the expression data (Table S1). The relative fold expressions of antioxidant enzymes encoding genes were measured by the comparative CT method [28].

### 2.11. Statistical Analysis

All the experiments were performed with seven biological-replicates, and each (biological repeats) had three plants (a total of 21 plants were used for each line per stress and per experiment; 7 × 3). The quantitative data were used to determine statistical significance by the analysis of variance among the mean values using Tukey’s HSD test and represented as mean ± SE (standard error). A significant difference among transgenic lines compared to control (WT and VC) plants under different stress conditions was considered at *p* < 0.05 and denoted by different lowercase letters.

## 3. Results

### 3.1. SbERD4 Is a Membrane-Localized Protein

**Figure 1:**
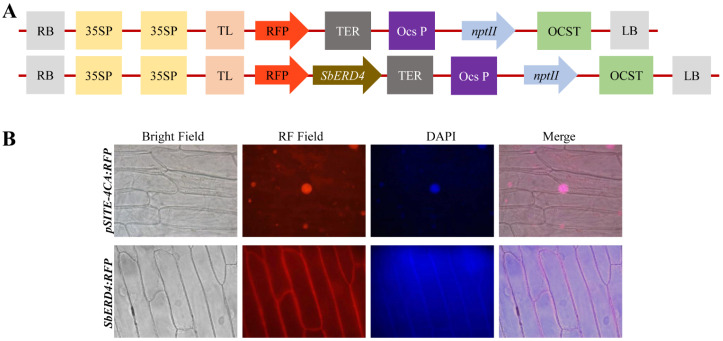
Subcellular localization study of *SbERD4* protein. (**A**) Schematic illustration of vectors *pSITE-4CA:RFP* and gene construct *SbERD4:RFP*, and (**B**) Onion epidermal cells transformed with *SbERD4:RFP* fusion construct and vector alone (*pSITE-4CA:RFP*).

Onion epidermal cells transformed with *SbERD4:RFP* fusion construct displayed extensive fluorescence signals in the plasma membrane, whereas cells transformed with vector alone (pSITE-4CA:RFP) exhibited a uniform dispersal of fluorescence all over the cell (Figure 1). The short-term expression of the *SbERD4:RFP* construct revealed that *SbERD4* is localized in the plasma membrane. In silico analysis predicted that the *SbERD4* protein is comprised of a transmembrane domain that showed resemblance with the Ca-dependent channel. Furthermore, in silico analysis showed that the protein has 10-transmembrane domains (Supplementary Figure S1).

### 3.2. SbERD4 Gene Expresses Differentially under Different Abiotic Stress Conditions

The real-time quantitative PCR (qRT-PCR) analysis showed the differential expression (up-regulation) pattern of the *SbERD4* gene in *S. brachiata* under different abiotic stress conditions (Supplementary Figure S2). The maximum 20, 15, and 10-fold relative expression of the *SbERD4* gene was observed under salt (500 mM NaCl), drought/osmotic (300 mM mannitol), and heat (40 °C) stress treatments at 24 h. However, a maximum of 16-fold expression was noticed at 3 h under cold (4 °C) stress conditions. The relative fold expression of the gene declined at 48 h under all abiotic stress treatments.

### 3.3. Cloning, Genetic Transformation, and Expression Analyses

The cDNA sequence of *SbERD4* is 2367 bp (GenBank no. MK629705), which is comprised of a 5’ untranslated region (5′–UTR: 1–64 bp), an open reading frame (ORF: 65–2191 bp), and 3′-UTR (2192–2367 bp). The *SbERD4* encodes for CSC1-like ERD4 protein (708 amino-acids; accession no. QFG71781).

**Figure 2:**
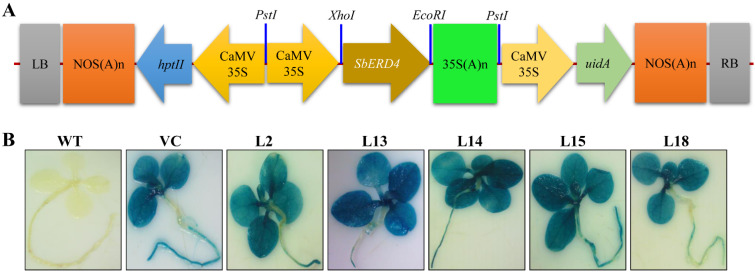
Gene construct and histochemical GUS assay. (**A**) Schematic representation of *pCAMBIA1301::35SSbERD4* gene construct, and (**B**) histochemical GUS assay of transgenic lines (L2, L13, L14, L15, and L18) and control plants (WT and VC).

A gene cassette *pCAMBIA1301::35SSbERD4* (Figure 2) was transformed to the tobacco plant, and about thirty putative transgenic lines (T0) were raised. Seeds of T0 transgenic lines were germinated on hygromycin, and twenty-one transgenic (T1) plants were raised. Transgenic plants were further validated by the amplification of *hptII* (0.96 kb) and *uidA* (1.2 kb) genes using PCR (Supplementary Figure S3). Semi-quantitative reverse transcriptase PCR showed the higher expression of transgenic lines L2, L13, L14, L15, and L18 compared to others, and therefore, they were selected for functional characterization. Additionally, these five transgenic (L2, L13, L14, L15, and L18) lines (Figure 2) also exhibited higher gus-expression.

### 3.4. Overexpression of the SbERD4 Gene Improves Plant Growth under Stress Conditions

About 83–85% seed germination was observed for control (VC and WT) and transgenic (L2, L13 L14, L15, and L18) plants under normal (unstressed) condition. Transgenic (L2, L13 L14, L15, and L18) had a significant (*p* < 0.05) higher seed-germination percentage (about 70%) compared to controls (VC and WT) (40–45%) under salinity (200 mM NaCl) or drought/osmotic (300 mM mannitol) stress (Supplementary Figure S4).

**Figure 3:**
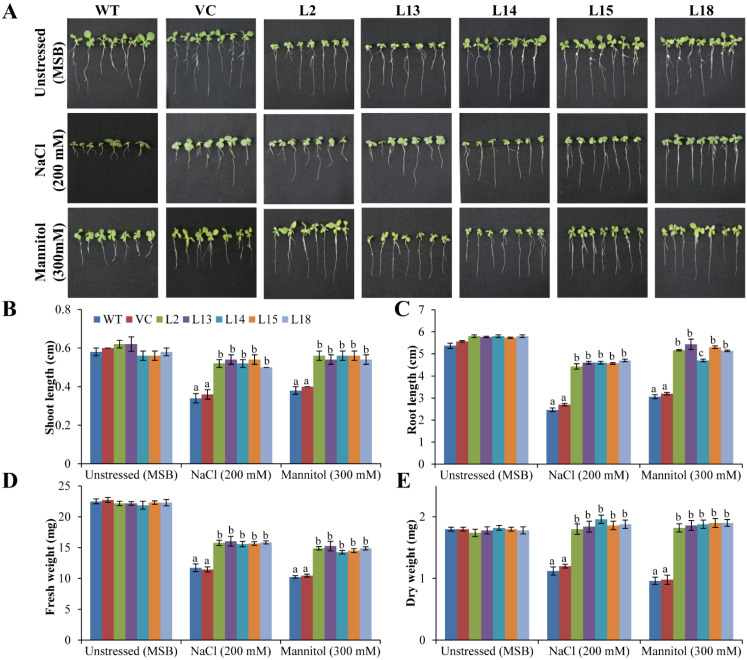
Plant growth analysis under abiotic stress conditions. Comparative analyses of (**A**) morphology, (**B**) shoot length, (**C**) root length, (**D**) fresh weight, and (**E**) dry weight of transgenic lines (L2, L13, L14, L15, and L18) and control plants (WT and CV) grown under unstressed (MSB), salt (200 mM NaCl), and drought/osmotic (300 mM mannitol) stress conditions. Data shown as mean ± SE and values shown with different letters are significant at *p* < 0.05.

All plants showed comparable growth parameters, including SL (0.6 cm), RL (5–6 cm), Fw (22 mg), and Dw (2 mg) under normal (unstressed) condition. Transgenic plants showed significantly (*p < 0.05*) higher growth parameters: SL (0.5–0.6 cm), RL (4.5–5.0 cm), Fw (15–16 mg), and Dw (1.8–1.9 mg) compared to controls (VC and WT) (SL: 0.35 cm, RL: 2.5–3.0 cm, Fw: 10–11 mg, and Dw: 1.0 mg) under salt or drought/osmotic stress (Figure 3). Transgenic leaves showed lessened chlorosis and necrosis compared to control plants and their respective counterparts (grown in normal conditions) under salinity or drought/osmotic stress conditions (Supplementary Figure S5). Furthermore, transgenic lines exhibited a high content of photosynthetic pigments (total chlorophyll, chlorophyll a, and chlorophyll b) compared to controls under different stress conditions (Supplementary Figure S5). The results indicate that the overexpression of the *SbERD4* enhanced the seed germination and plant growth of transgenic plants under varying stress conditions.

### 3.5. Ectopic Expression of the SbERD4 Gene Augments the Physiological Status of Plants under Different Abiotic Stress Conditions

**Figure 4:**
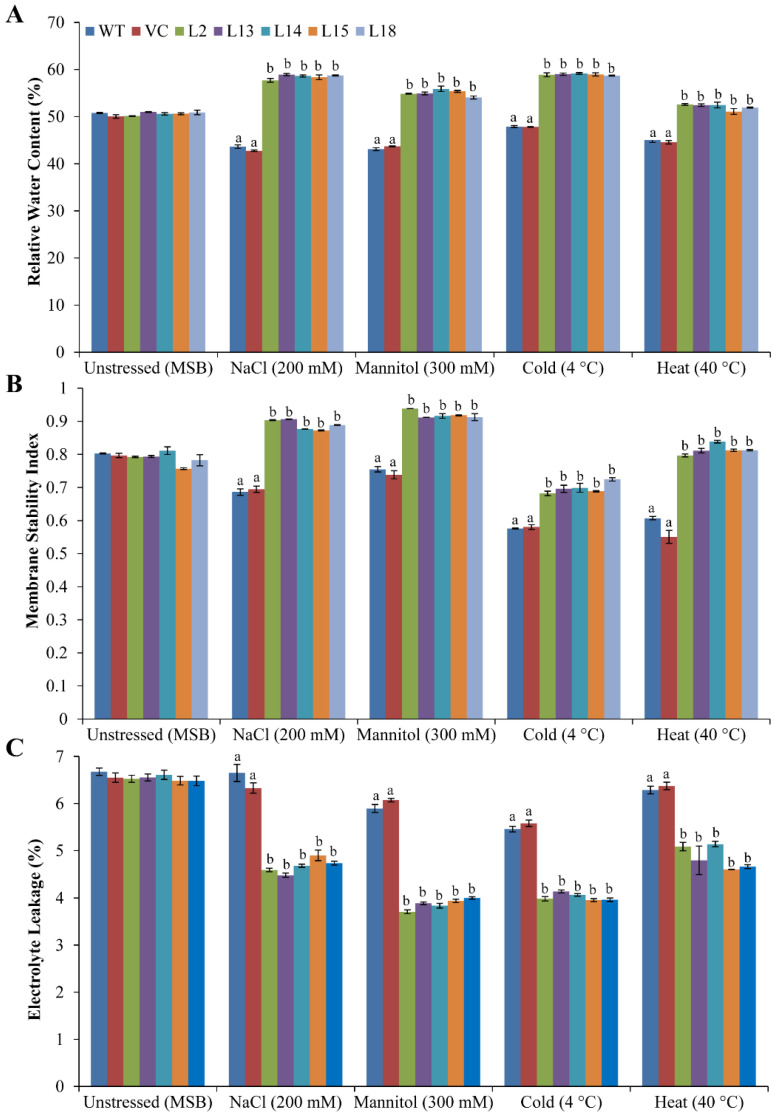
Analysis of plant physiology under diverse abiotic stress conditions. Comparative analyses of (**A**) relative water content (RWC), (**B**) membrane stability index (MSI), and (**C**) electrolyte leakage (EL) of transgenic lines (L2, L13, L14, L15, and L18) and control plants (WT and CV) grown under unstressed (MSB), salt (200 mM NaCl), osmotic (300 mM mannitol), cold (4 °C), and heat (40 °C) stress conditions. Data are shown as mean ± SE and different letters show significance at *p* < 0.05.

Distinct physiological indicators (RWC, EL, and MSI) were calculated and compared with control plants under various stress conditions (Figure 4). About 50% RWC was found for all plants grown in normal (unstressed) conditions; transgenic showed high RWC under salt (58–60%), osmotic (54–55%), cold (59%), and heat (51–52%) stress conditions compared to control (WT and VC) plants (43–48%). Similarly, high MSI (0.9 for salinity and osmotic; 0.7–0.8 for cold and heat) was found for transgenic lines compared to control plants (0.6–0.7) under different stress conditions. However, all plants showed about 0.8 MSI under unstressed conditions. In contrast, low EL was observed for transgenic lines (4–5%) compared to control plants (6–7%) under stress treatments. However, about 6.5% EL was noticed under unstressed conditions. The results (Figure 4) unveiled that overexpression of the *SbERD4* gene enhanced the physiological status of transgenic by increasing RWC and MSI and lowering EL under stress conditions compared to controls.

### 3.6. Osmoprotectants Accumulate in Plants Overexpressing the SbERD4 Gene under Stress Conditions

**Figure 5:**
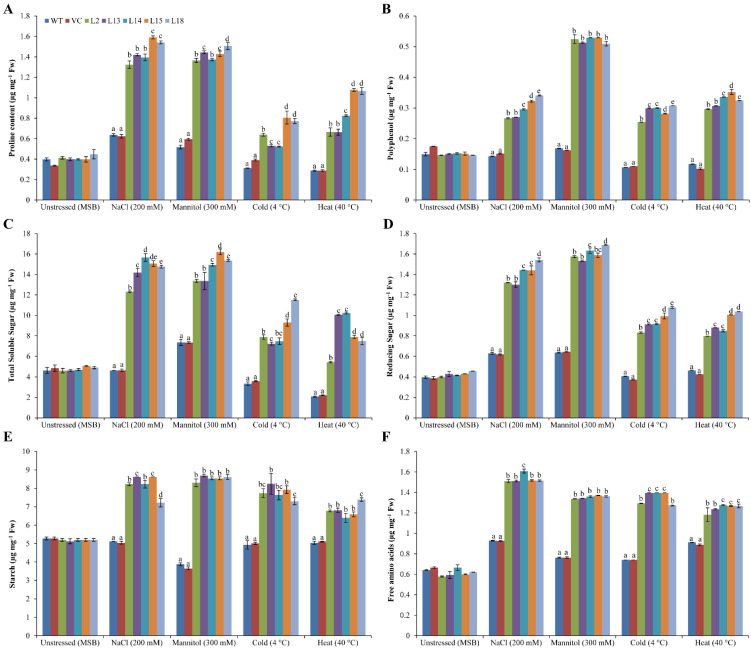
Quantification of osmoprotectant contents under diverse abiotic stress conditions. Comparative analyses of (**A**) proline, (**B**) polyphenol, (**C**) total soluble sugar (TSS), (**D**) reducing sugar (RS), (**E**) starch, and (**F**) free amino acid (FAA) contents of transgenic (L2, L13, L14, L15, and L18) and control plants (WT and CV) grown under unstressed (MSB), salt (200 mM NaCl), osmotic (300 mM mannitol), cold (4 °C), and heat (40 °C) stress conditions. Data are shown as mean ± SE and different letters show significance at *p* < 0.05.

The concentration of osmoprotectants, including proline, polyphenols, total sugars, reducing sugars, starch, and free amino acids was about 0.4, 0.15, 5.0, 0.4, and 0.65 µg mg^−1^ Fw, respectively, among transgenic lines and control (WT and VC) plants under unstressed condition (Figure 5). Enhanced proline concentration was detected in transgenic plants under stress (salt/osmotic: 1.4–1.6 and cold/heat: 0.5–1.0 µg mg^−1^ Fw) conditions compared to control plants (0.5–0.6 in salt/osmotic and 0.3–0.4 in cold/heat). High polyphenols accumulation was observed in transgenic lines (salt/osmotic: 0.3–0.5 and cold/heat 0.25–0.35 µg mg^−1^ Fw) under stress conditions compared to control plants, in which no change was noticed. The high accumulation of total soluble sugars was found in the transgenic lines under stress (salt/osmotic: 12–16 and cold/heat: 7–11 µg mg^−1^ Fw) conditions compared to control plants (salt/osmotic: 5–7 and cold/heat: 2–3.5 µg mg^−1^ Fw). Similarly, a high content of reducing sugars and free amino acids were detected in transgenic lines (salt/osmotic: 1–1.6 and cold/heat: 0.8–1.4 µg mg^−1^ Fw) under stress compared to control plants in which negligible changes in the concentration were noticed. No change in the starch content was found in control plants under stress conditions; however, high content was detected in transgenic lines (7–8 µg mg^−1^ Fw). The results exhibited that overexpression of the *SbERD4* gene leads to the accumulation of an osmoprotectant to cope with the detrimental effect of abiotic stress conditions (Figure 5).

### 3.7. The SbERD4 Gene Involved in Ion Homeostasis Maintenance

The transgenic plants overexpressing the *SbERD4* gene showed less Na^+^ accumulation and higher K^+^ accumulation compared to controls (VC and WT) under salinity stress conditions. About 0.05 and 0.1 mg g^−1^ Dw Na and K ions, respectively, were estimated in all plants under unstressed conditions (Supplementary Figure S6). High Na^+^ content and about 0.2 mg g^−1^ Dw was found in control plants, while there was an insignificant (*p* > 0.05) change in the Na^+^ content (0.06–0.08 mg g^−1^ Dw) in transgenic lines under the salt stress condition. In contrast, transgenic plants showed a higher concentration of K^+^ (0.25 mg g^−1^ Dw) as compared to control plants (0.01 mg g^−1^ Dw) under salt stress conditions (Supplementary Figure S6). Moreover, transgenic plants also showed a considerably lower Na^+^/K^+^ (i.e., higher K^+^/Na^+^) ratio under exposure to salinity. Herein, the concentration of potassium and sodium as well as the K^+^/Na^+^ ratio were comparable in transgenic and control plants (VC and WT).

### 3.8. ROS Buildup and Oxidative Damage Mitigated by the Overexpression of SbERD4 Gene

In the control condition, the H_2_O_2_ content was comparable in transgenic lines and control plants under the unstressed condition (Supplementary Figure S7). *SbERD4* overexpressing transgenic lines showed considerably (*p* < 0.05) low accumulation of H_2_O_2_ contents compared to control plants under different stress conditions. Transcript expression analysis of ROS scavenging antioxidant enzyme encoding genes such as *NtAPX, NtSOD, NtCAT*, and *NtGR* showed that their expression was comparable in all plants under unstressed conditions. However, their expression was up-regulated many folds (2–6 fold) under different abiotic stress conditions compared to control (VC and WT) plants (Supplementary Figure S8). The expression of superoxide dismutases (SOD) and ascorbate peroxidase (APX) encoding genes were up-regulated 3–5 fold, catalase (CAT encoding gene 2–6 fold, while the glutathione reductase (GR) encoding gene was up-regulated 2–4 fold in transgenic plants under stress conditions (Supplementary Figure S8). These results explained that the ectopic expression of the *SbERD4* gene may regulate the expression of genes that encode for antioxidant enzymes and thus enhanced the ROS scavenging mechanism of transgenic plants under stress.

### 3.9. SbERD4 Overexpressing Transgenic Lines Perform Better under Stress Conditions

**Figure 6:**
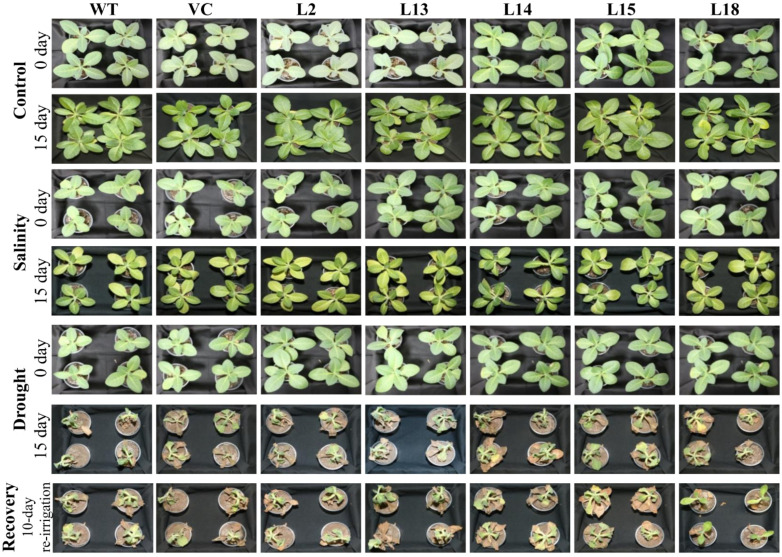
A comparative plant growth analysis. Morphological analysis of transgenic lines (L2, L13, L14, L15, and L18) and control plants (WT and CV) grown under different (control, salinity, and drought) conditions.

In order to evaluate the growth of transgenic plants overexpressing the *SbERD4* gene in greenhouse conditions, twenty-eight-day-old plants (L2, L13 L14, L15, L18, WT, and VC) of equal size were progressively subjected to salt or drought/osmotic stress. Transgenic plants showed better growth compared to control plants, while non-transgenic showed retarded growth (Figure 6). Furthermore, non-transgenic plants recovered slowly when normal (unstressed) condition was applied compared to transgenic plants, which gain healthy growth easily (Figure 6).

## 4. Discussion

Amongst the major abiotic stresses, salt, osmotic/drought, and cold adversely affect plants’ growth, limiting their productivity for the ever-growing world population. An adaptive mechanism has been evolved by plants to endure the malicious effects of different abiotic stresses. Commonly, plants modulate their several metabolic and physiological processes to cope with these conditions. Indeed, many stress-related genes were characterized from halophytes, and novel candidate genes are always welcomed, which provide endurance for a sustainable demand of agriculture [8,11,42]. In this study, a novel CSC1-like gene *SbERD4* was isolated from a nutritionally and industrially important extreme halophyte *Salicornia brachiata* [43,44,45,46], and it was functionally characterized into *Nicotiana tabacum* cv. Petit Havana through a transgenic approach.

In this study, *SbERD4* overexpressing transgenic plants were adapted to withstand water-deficit conditions to maintain normal physiological processes. Indeed, plant drought tolerance is a complex phenomenon, with many attributes included in the drought stress, and the plant may have managed to respond to this signal in different ways [47]. Plants respond to drought stress in several ways such as drought avoidance (closing of stomata, growing deeper roots, and depositing wax in leaves), drought escape (shorting life cycle), and drought tolerance (generation of antioxidant, osmolytes, and some stress-relieving agent). In this study, *SbERD4* overexpressing transgenic lines exhibited greater tolerance to drought stress, producing suitable osmolytes, higher free radicles scavenging enzyme, and improved several physiobiological activities as a reference to the control plant. Other studies corroborate that the overexpression of *SpERD15* confers multiple abiotic stress tolerance in tobacco [48], whereas ERD7 gene expression is induced by different abiotic stress conditions [49].

High expression of the *SbERD4* gene was found under drought (osmotic/desiccation), salt, and cold stress conditions (Supplementary Figure S2). Parallel to this result, an improved abiotic stress tolerance was reported in *Salicornia* due to the high expression of various abiotic stress-responsive genes [12,50,51]. It is well known that salinity has a strong impact on the gene expression. Salt-responsive genes directly protect cells from stress or by encoding regulatory proteins that are involved in the synthesis of detoxification enzymes, osmoprotectants, transporters, and ion channels [52].

The *SbERD4* gene sequence showed 79.75% identity with *Spinacea oleracea*, 79.60% identity with *Beta vulgaris*, and 78% identity with *Chenopodium quinoa*, predicted CSC1-like ERD4 protein. Subcellular localization studies are commonly performed to predict the function of a protein. Transient expression revealed that the *SbERD4* gene localized in the plasma membrane (Figure 1), which was further supported by in silico analysis. Rai et al. [53] stated that ERD4 is a trans-membrane protein, whose role may play an important role in improving abiotic stress tolerance in plants. Plants have a number of plasma membrane-bound proteins, and transport protein are very common, which help in the cross-talk at the interface and also sensitize plants to adjust themselves in the changing environment [30,54,55].

When the *SbERD4* overexpressing transgenic lines were subjected to different abiotic stresses, an enhanced tolerance against these stresses was observed in the T1 generation. The *SbERD4* overexpressing transgenic lines were found to have a high seed germination percentage compared to control plants under osmotic/drought and salinity conditions. Five transgenic lines—L2, L13 L14, L15, and L18—showing high seed germination and also GUS assay were selected to functionally validate the *SbERD4* gene in various abiotic stress tolerances (Figure 2 and Supplementary Figure S4). It is proclaimed that chlorophylls generally degraded in abiotic stresses because of the excess generation of ROS in the chloroplast [56]. Thus, chlorophyll is considered as a cellular marker for stress. The transgenic lines raised in the study did not show significant degradation of chlorophylls in abiotic stress conditions, and therefore, leaf damages were not found in transgenic plants compared to controls (Supplementary Figure S5). Plant senescence depends on the integrity of cellular components; cell loose compartmentalization and tissue structure in plants lead to death [57]. Transgenic plants were observed to tolerate the elevated salt and drought/osmotic stress by leaf disc senescence assay (Supplementary Figure S5) compared to control plants [17,31,33]. Similarly, transgenic plants overexpressing *SbASR1* and *SbAPX* genes were reported to contain high chlorophyll under drought/osmotic and salinity stress conditions [58,59,60]. The protection of chlorophyll pigments from oxidative damage is essential for sustainable photosynthesis under abiotic stresses, and the results demonstrated that *SbERD4* overexpressing transgenic lines did it very well.

Improved plant growth including root length, shoot length, fresh weight, and dry weight was noticed in transgenic plants under different stress conditions such as salinity, drought, cold, and heat compared to control plants (Figure 3). The enhanced biomass of transgenic lines confirmed the remarkable features of the *SbERD4* gene in reducing the adverse effect of stress and also providing tolerance to the plant under a range of different abiotic stress conditions. RWC, EL, and MSI (Figure 4) confirmed the physiological status of plants, and enhanced stress tolerance of transgenic (*SbERD4*) was achieved by modulating the physiology of plants. Significantly high MSI and RWC were found in transgenic plants under both unstressed and stressed conditions. Consistent with our results, a number of related reports showed that the transgenic model plant (tobacco) transformed with novel genes including *SbUSP*, *SbSRP*, *SbCDR*, and *SbSDR1* exhibited similar physiology under different stress conditions [30,31,50,55].

Abiotic stress can perturb the osmotic balance of plants. In order to cope with different stresses, plants accumulated various osmoprotectants such as sugar, proline, and polyphenol, which are considered cellular stress markers [61]. The accumulation of these substances protects the cells by reducing the osmotic pressure of the cell in plants, which maintains the osmotic potential of the cell membrane under osmotic/drought stress [56]. Transgenic (*SbERD4*) lines showed a higher accumulation of osmoprotectants such as total sugar, proline, free amino acids, and polyphenol than controls under different stress conditions (Figure 5). Similar results were also reported in transgenic plants that overexpressed different abiotic stress-responsive genes under various stress conditions [30,31,55]. The accumulation of proline in the cells acts as an osmoprotectant, and it acts as a molecular chaperone and ROS scavenger to protect other macromolecules to maintain the cell stability [30]. Furthermore, the extensive accumulation of proline is an indicator of osmotic tolerance, and soluble sugars can maintain the structure and function of macromolecules [62]. Polyphenol accumulation contributes to defense against reactive oxygen species [63]. Free amino acids act as building blocks in the biosynthesis of different proteins and thus serve as signaling and/or regulatory molecules [64]. In the study, it was observed that a higher accumulation of osmoprotecrtans improved the physiology of plants under different stress conditions including salinity, drought/osmotic, cold, and heat.

A high Na^+^ content triggers detrimental effects on cell metabolism, decreases the photosynthetic performance in plants, and causes oxidative damage [65]. Under stress conditions, *SbERD4* transgenic plants maintained a high concentration of K^+^ and K^+^/Na^+^ ratio and accumulated a low level of Na^+^ compared to the controls (Supplementary Figure S6). In agreement with these results, the overexpression of different abiotic stress-related genes showed a similar pattern of results under stress conditions [31,50,55]. It was observed that *SbERD4* may provide stress tolerance by maintaining the ionic balance across the cell under stress.

In this study, a lower accumulation of H_2_O_2_ was found in transgenic plants under unstressed and stressed conditions in comparison to control plants (Supplementary Figure S7). Similar to the previous studies [55,60], the results suggested the role of the *SbERD4* gene in improving oxidative stress by improving ROS scavenging activity. In general, abiotic stresses are an after-effect of the interruption between ROS production and anti-oxidant defense in plants [66]. Stress tolerance is commonly associated with the augmented anti-oxidant defense systems of the plant [67]. It is stipulated that anti-oxidant defense systems depend on the intensity of stress and the different developmental stages of plants [68]. It is elucidated that ROS is generally produced in a plant under stress and leads to oxidative damages. ROS scavenger enzyme activity plays a key role in maintaining cell homeostasis by exploring the decomposition and detoxification process mediated by different antioxidant enzymes [69]. In the study, the higher expression of f *NtSOD*, *NtAPX, NtGR*, and *NtCAT* genes under drought, salt, cold, and heat stress revealed the high activity of the corresponding antioxidant enzymes in transgenic plants compared to the respective controls (Supplementary Figure S8). Furthermore, the results suggested a possible role of *SbERD4* in the ROS-scavenging mechanism. The gene *SbERD4* may directly or indirectly regulate the ROS scavenging by controlling the activity of antioxidant enzymes through their gene expression [70].

*SbERD4* transgenic and control plants were gradually exposed to salinity and osmotic/drought in a controlled culture condition to evaluate their growth performance in a possible natural environmental condition [71]. The pattern of gene expression and abiotic stress tolerance of a plant is quite different. Plant tolerance depends on the different growth stages under a particular environmental condition, and it defers from one growth stage to another. *SbERD4* transgenic lines showed enhanced growth under normal (unstressed) condition. Furthermore, transgenic plants were less affected when gradually exposed to stress, whereas the WT and VC plants were severely affected (Figure 6).

## 5. Conclusions

*SbERD4* is a novel dehydration stress protein characterized from extreme halophytes *Salicornia brachiata*. Our finding demonstrated that the *SbERD4* gene is localized in the plasma membrane, and its function is to improve stress tolerance in plants under different abiotic stress conditions. The results also suggest that the *SbERD4* gene might play a key role in maintaining an osmotic tolerance against multiple abiotic stress conditions through evaluating their function under various physiological and biochemical parameters. Taken together, these results suggest that the *SbERD4* gene might be used as a promising candidate gene for developing stress-tolerant crop plants. Further studies are required to understand the unprecedented information related to the interaction between *SbERD4* genes and other promising genes that can unveil the salinity and osmotic stress tolerance in crop plants.

## Data Availability

Sequence data are available at NCBI (https://www.ncbi.nlm.nih.gov) with accession numbers: MK629705 and QFG71781.

## Supplementary Materials

The following are available online at https://www.mdpi.com/article/10.3390/cells11010062/s1, Table S1: Primers and PCR conditions used in the study, Figure S1: In silico analysis of *SbERD4* protein. (**A**) *SbERD4* protein resembles the Ca-dependent channel, and (**B**) the *SbERD4* protein comprises 10-transmembrane domains, Figure S2: Transcript expression analysis of the *SbERD4* gene. Relative expression of the *SbERD4* gene was analyzed under different abiotic stress treatments; salinity (500 mM NaCl), drought (300 mM mannitol), cold (4 °C), and heat (40 °C) up to 48 h (3, 6, 12, 24, and 48 h), Figure S3: Molecular confirmation of putative transgenic lines. Agarose gels showing PCR amplification of (**A**) *hptII* and (**B**) *uidA* genes. Lane M: marker, Lane 1: Positive control (i.e., gene construct *pCAMBIA1301::35SSbERD4* used as templet), Lane 2: Negative control (i.e., DNA from WT used as templet), Lane 3–16: Randomly selected putative transgenic lines, Figure S4: Seed germination analysis. Seeds of *SbERD4* overexpressing transgenic lines (L2, L13, L14, L15, and L18) and control plants (WT and VC) were germinated on (**A**) MSB (Murashige & Skoog Basal) media supplemented with NaCl (200 mM) or mannitol (300 mM) for (**B**) percentage seed-germination analysis. Bars represent means ± SE, and values with different letters are significant at *p* < 0.05, Figure S5: Leaf senescence and chlorophyll content analysis. The senescence assay (**A**) showed reduced chlorosis and less damage (necrosis) in transgenic plants compared to control plants and unstressed conditions. Further transgenic lines showed high (**B**) total chlorophyll, (**C**) chlorophyll a, and (**D**) chlorophyll b under salinity and osmotic stress conditions. Bars represent means ± SE, and values with different letters are significant at *p* < 0.05, Figure S6: Ion content analysis. (**A**) Na ion, (**B**) K ion, and (**C**) Na^+^/K^+^ ratio were estimated in the transgenic plants under salt stress conditions and compared with control plants. Bars represent means ± SE, Figure S7: Analysis of H_2_O_2_ content. Quantitative analyses of H_2_O_2_ content of transgenic lines (L2, L13, L14, L15, and L18) and control plants (WT and CV) grown under unstressed (MSB), salt (200 mM NaCl), osmotic (300 mM mannitol), cold (4 °C), and heat (40 °C) stress conditions. Bars represent means ± SE, and values with different letters are significant at *p* < 0.05, Figure S8: Transcript expression analysis of ROS-scavenging antioxidant enzyme encoding genes. The relative fold expression of (**A**) the *NtSOD* gene encoding superoxide dismutases, (**B**) *NtAPX* encoding ascorbate peroxidase, (**C**) *NtCAT* encoding catalase, and (**D**) *NtGR* encoding glutathione reductase enzyme was analyzed under different stress conditions.
