# Supplementary material for: Introgression of SbERD4 Gene Encodes an Early-Responsive Dehydration-Stress Protein That Confers Tolerance against Different Types of Abiotic Stresses in Transgenic Tobacco
Source: Cells. 2021 Dec 27;11(1):62. doi: 10.3390/cells11010062 (PMC8750158; doi:10.3390/cells11010062)
Supplement: Supplementary file 1 [file cells-11-00062-s001.zip › cells-1487248-supplementary.pdf]

Table S1: Primers and PCR conditions used in the study.

Purpose	Primer	Orientation	Sequence (5'-3')	PCR conditions
RACE (Rapid Amplification of cDNA Ends)	GSP5-C38R1	Reverse	AAGTAGACCGCACCAAATG	As suggested 5' RACE System for Rapid Amplification of cDNA Ends, version 2.0
	GSP5-C38R2	Reverse	CCATGCTTCTTTCACTTCAG	
	GSP5-C38R3	Reverse	AATGGCAAGTGTCACAGCAA	
	GSP5-C38R4	Reverse	AACTCCAAACCATCCTCG	
	GSP5-C38R5	Reverse	ACGAATGCAAAGATCAACG	
	GSP5-C38R6	Reverse	CCATGGCTTCTTTCACTTCAG	
Full length, semi quantitative Rt PCR and confirmation of transgenic lines	ERD4-F	Forward	ATGGATTTCAGTTCGTTTGTA	95 °C, 5 min; 35 Cycles: 95 °C, 50 s; 55 °C, 30 s; 72 °C, 45 s; Extension: 72 °C, 7 min; 4 °C hold
	ERD4-R	Reverse	TTAAGCATCACCAACGTGAT	
Transcript profiling	ERD4RT-F	Forward	CGACAAGGTTTCCAGCTGA	95 °C, 3 min; 35 Cycles: 95 °C, 30 s; 55 °C, 30 s; 72 °C, 30 s; Extension: 72 °C, 10 min; 4 °C hold
	ERD4RT-R	Reverse	CACATCCTCCCATAGCTCT	
Cloning for subcellular localization study	ERD4SL-F	Forward	CACC ATGGATTTCAGTTCGTTTGTA	94 ºC, 5 min; 35 Cycles: 94 ºC, 30 s; 55 ºC, 30 s; 72 ºC, 30 s; Extension: 72 ºC, 10 min; 4 ºC hold
	ERD4SL-R	Reverse	TTAAGCATCACCAACGTGAT	
Cloning in pRT101 vector	ERD4pRT-F	Forward	CTCGAGAATGGATTTCAGTTCGTTTTTAAC	94 ºC, 5 min; 32 Cycles: 94 ºC, 30 s; 58 ºC, 40 s; 72 ºC, 50 s; 72 ºC, 10 min; 4 ºC hold
	ERD4pRT-R	Reverse	GGATCCTTAAGCATCACCAACGTGATCATA	
Expression analysis of antioxidant enzyme encoding genes by quantitative real time PCR	*Nt*APX-F	Forward	CAAATGTAAGAGGAAACTCAGAGGA	95 °C, 1 min; 40 Cycles: 94 °C, 10 s; 60 °C, 10 s; 72 °C, 15 s; Extension: 72 °C, 10 min; 4 °C hold
	*Nt*APX-R	Reverse	CAGCCTTGAGCCTCATGGTACCG	
	*Nt*CAT-F	Forward	AGGTACCGCTCATTCACACC	
	*Nt*CAT-R	Reverse	AAGCAAGCTTTTGACCCAGA	
	*Nt*SOD-F	Forward	AGCTACATACGCCTTTCC	
	*Nt*SOD-R	Reverse	CCCTGTAAAGCAGCACCTTC	
	*Nt*GR-F	Forward	ATAGATTCTGATGCTGCCCTTG	
	*Nt*GR-R	Reverse	CGACTCCTCAGTATGGAACTCAA	
Reference genes	*Sb*β-tubline-F	Forward	GGAGTCACCGAGGCAGAG	95 ºC, 5 min; 32 Cycles: 95 ºC, 50 s; 58 ºC, 45 s; 72 ºC, 1 min; Extension: 72 ºC, 7 min; 4 ºC hold
	*Sb*β-tubline-R	Reverse	ATCACATATCAGAAACCACAA	
	uidA-F	Forward	GATCGCGAAAACTGTGGAAT	
	uidA-R	Reverse	TGAGCGTCGCAGAACATTAC	
	*Nt*Actin-F	Forward	CGTTTGGATCTTGCTGGTCGT	
	*Nt*Actin-R	Reverse	CAGCAATGCCAGGGAACATAG	
	hptII-F	Forward	TTCTTTGCCCTCGGACGAGTG	
	hptII-R	Reverse	ACAGCGTCTCCGACCTGATG	

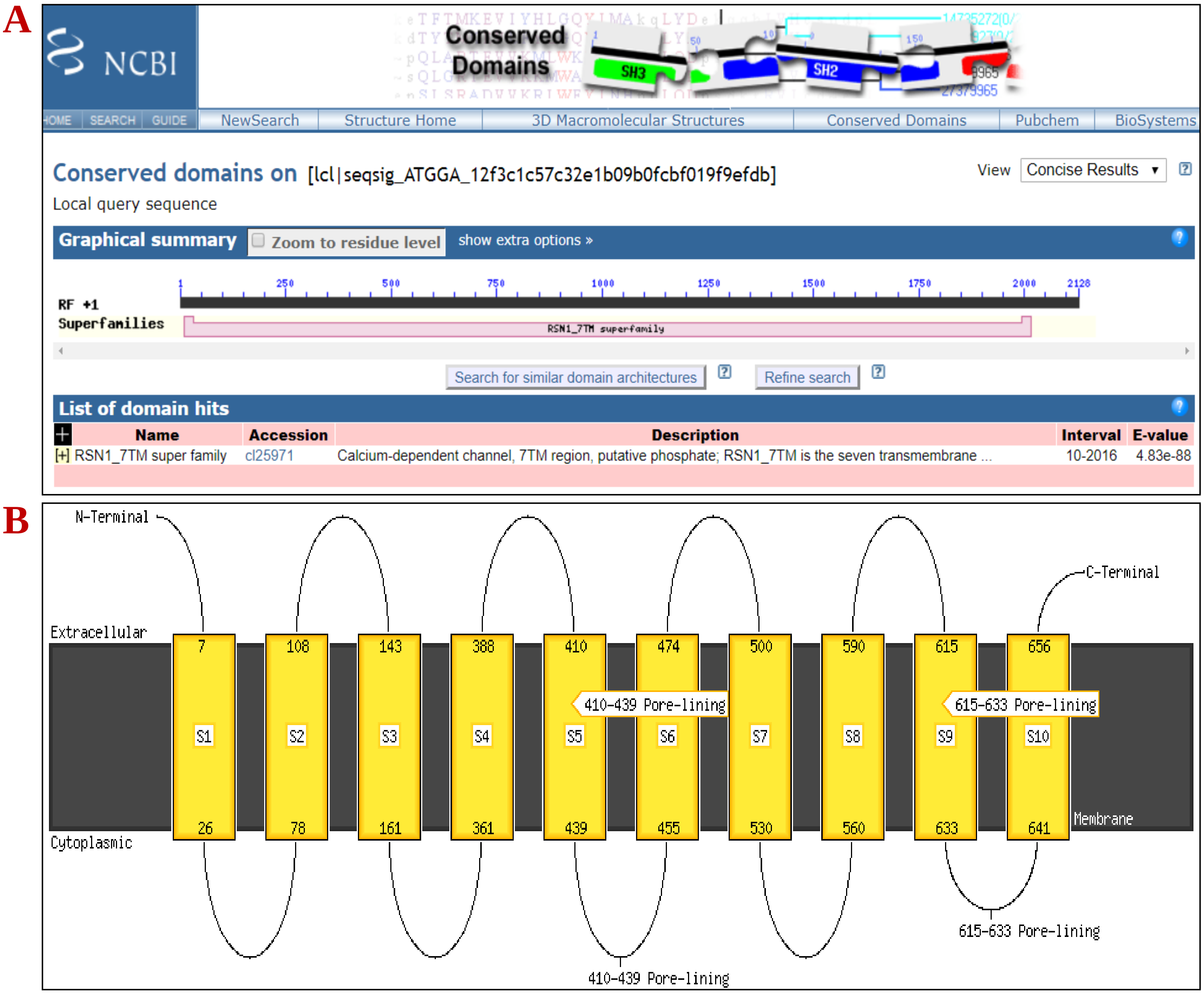

Figure S1: *In silico* analysis of *Sb*ERD4 protein. (**A**) *Sb*ERD4 protein resembles the Ca-dependent channel, and (**B**) the *Sb*ERD4 protein comprises 10-transmembrane domains

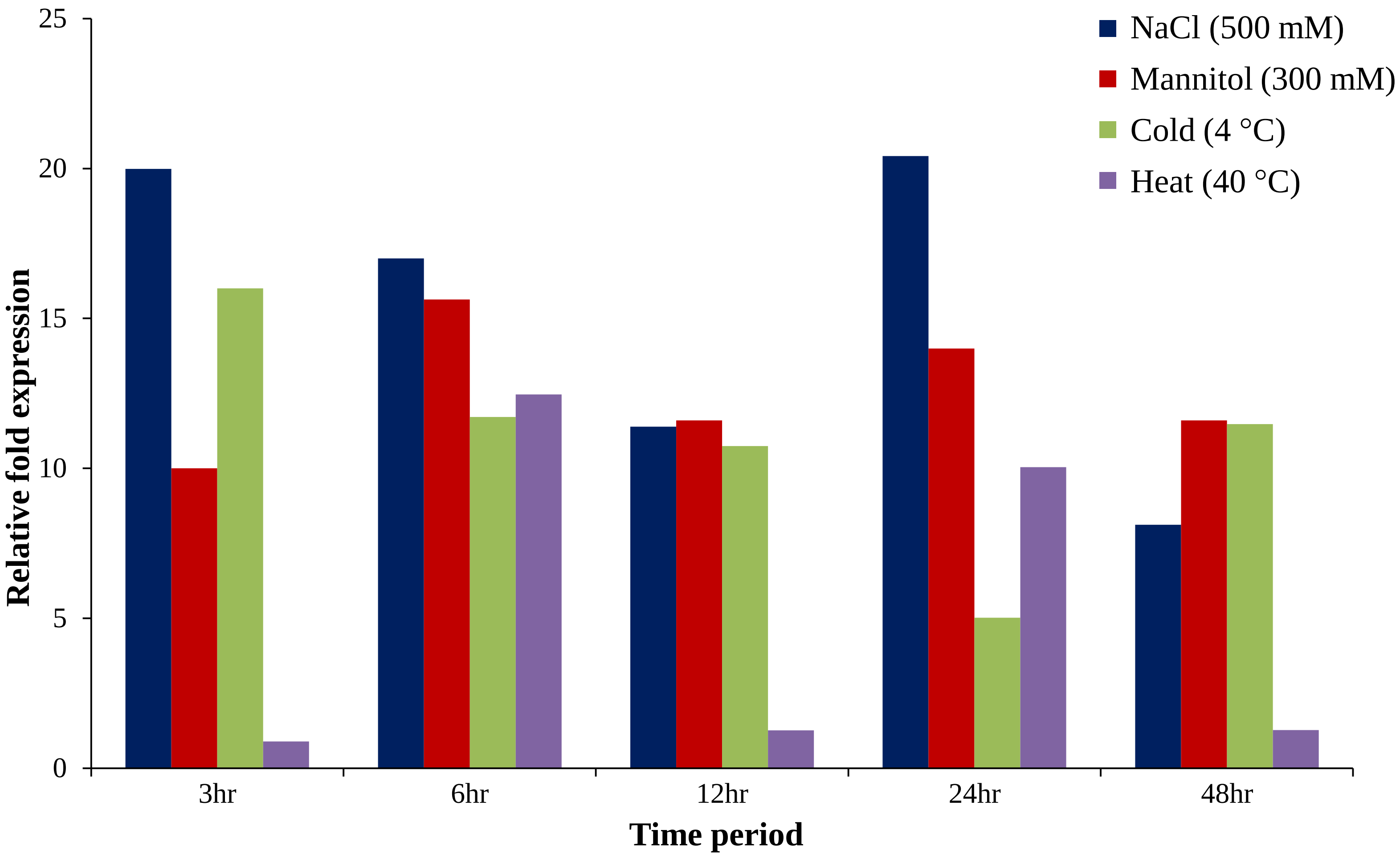

Figure S2: Transcript expression analysis of the *SbERD4* gene. Relative expression of the *SbERD4* gene was analyzed under different abiotic stress treatments; salinity (500 mM NaCl), drought (300 mM mannitol), cold (4 °C), and heat (40 °C) up to 48 h (3, 6, 12, 24, and 48 h).

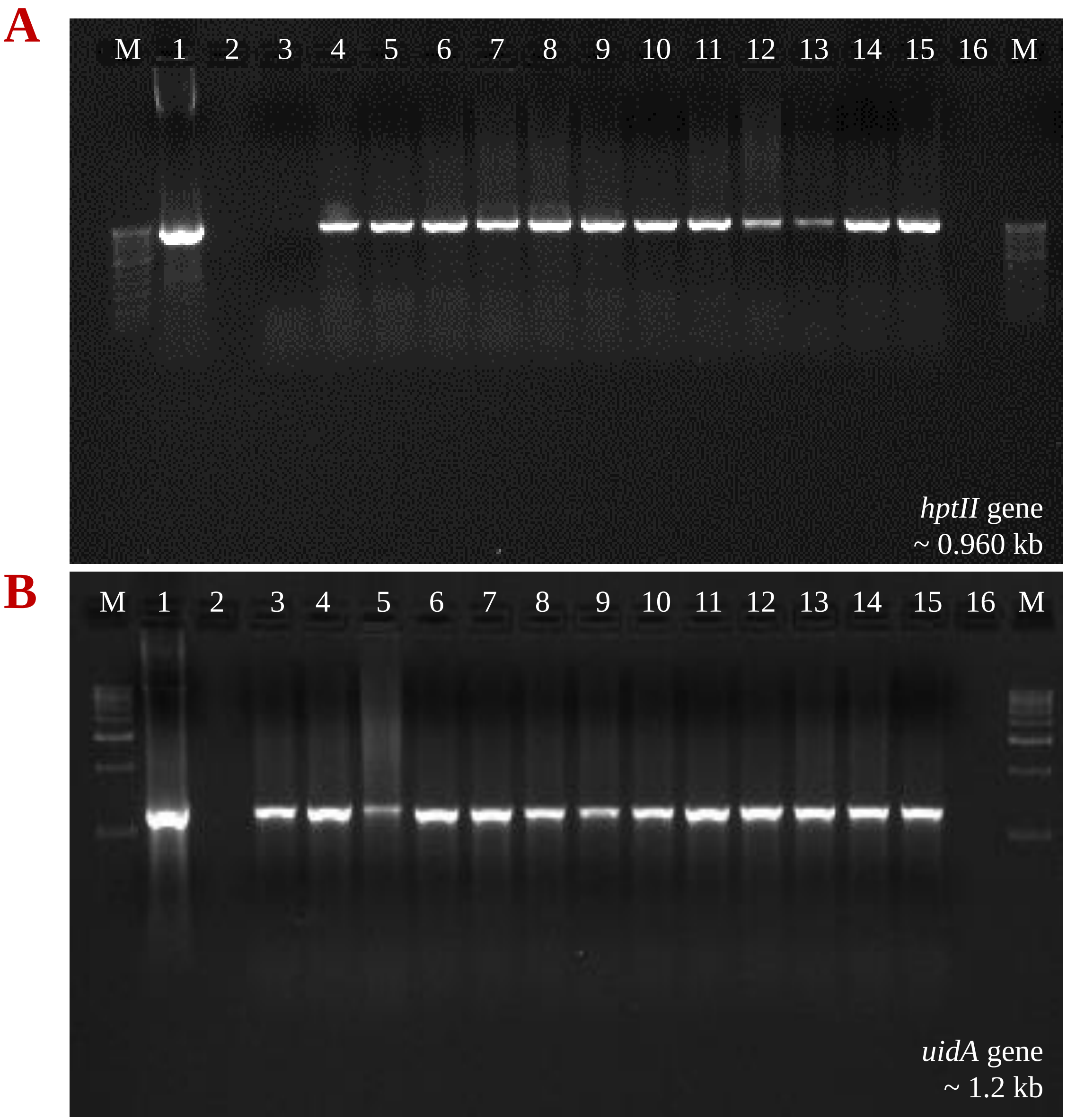

Figure S3: Molecular confirmation of putative transgenic lines. Agarose gels showing PCR amplification of **(A)** *hptII* and **(B)** *uidA* genes. Lane M: marker, Lane 1: +ve control (i.e. gene construct *pCAMBIA1301::35SSbERD4* used as templet), Lane 2: –ve control (i.e. DNA from WT used as templet), Lane 3–16: randomly selected putative transgenic lines.

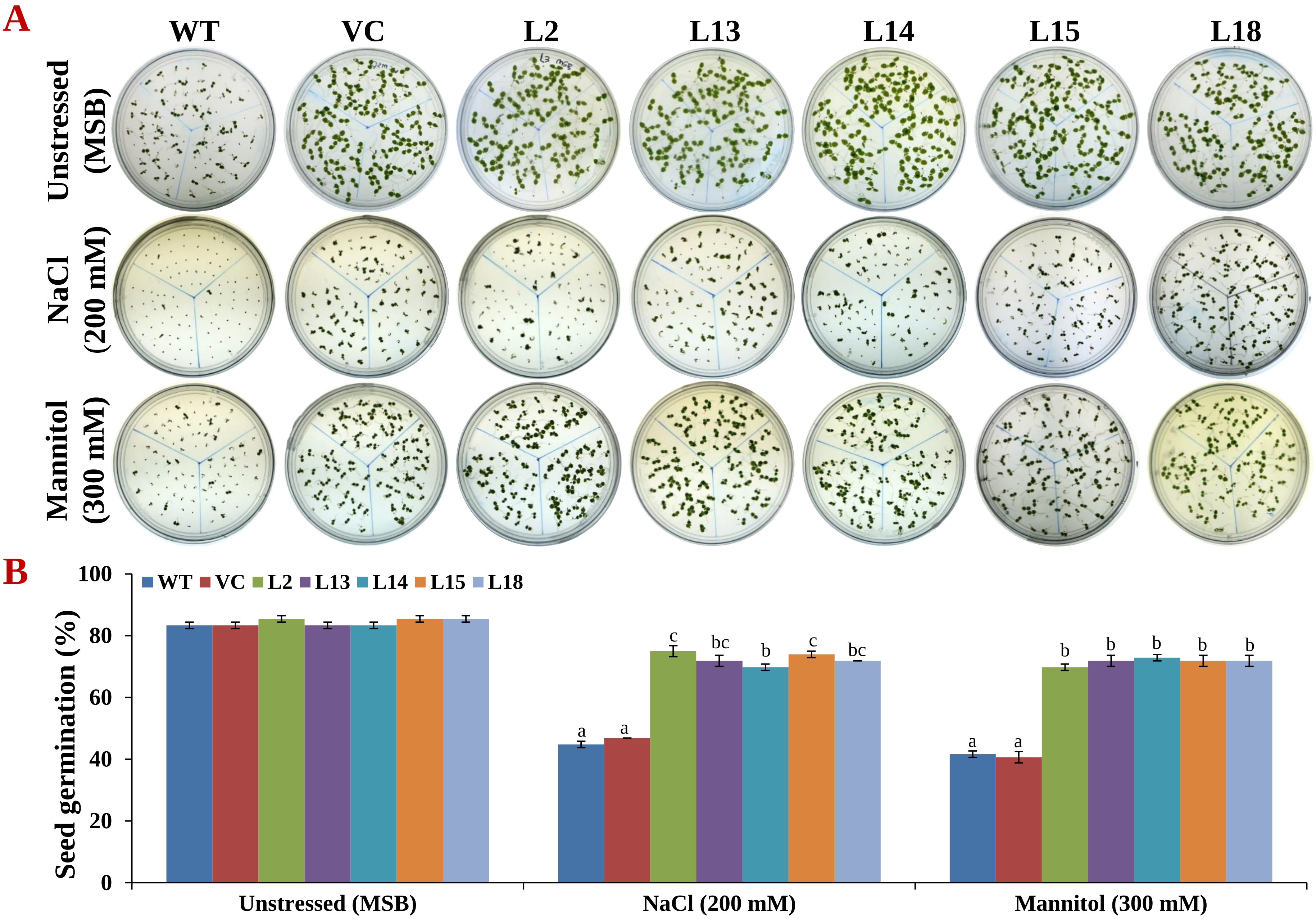

Figure S4: Seed germination analysis. Seeds of *SbERD4* overexpressing transgenic lines (L2, L13, L14, L15 and L18) and control plants (WT and VC) were germinated on (**A**) MSB (Murashige & Skoog Basal) media supplemented with NaCl (200 mM) or mannitol (300 mM) for (**B**) percentage seed-germination analysis. Bars represent means ± SE and values with different letters are significant at $p < 0.05$.

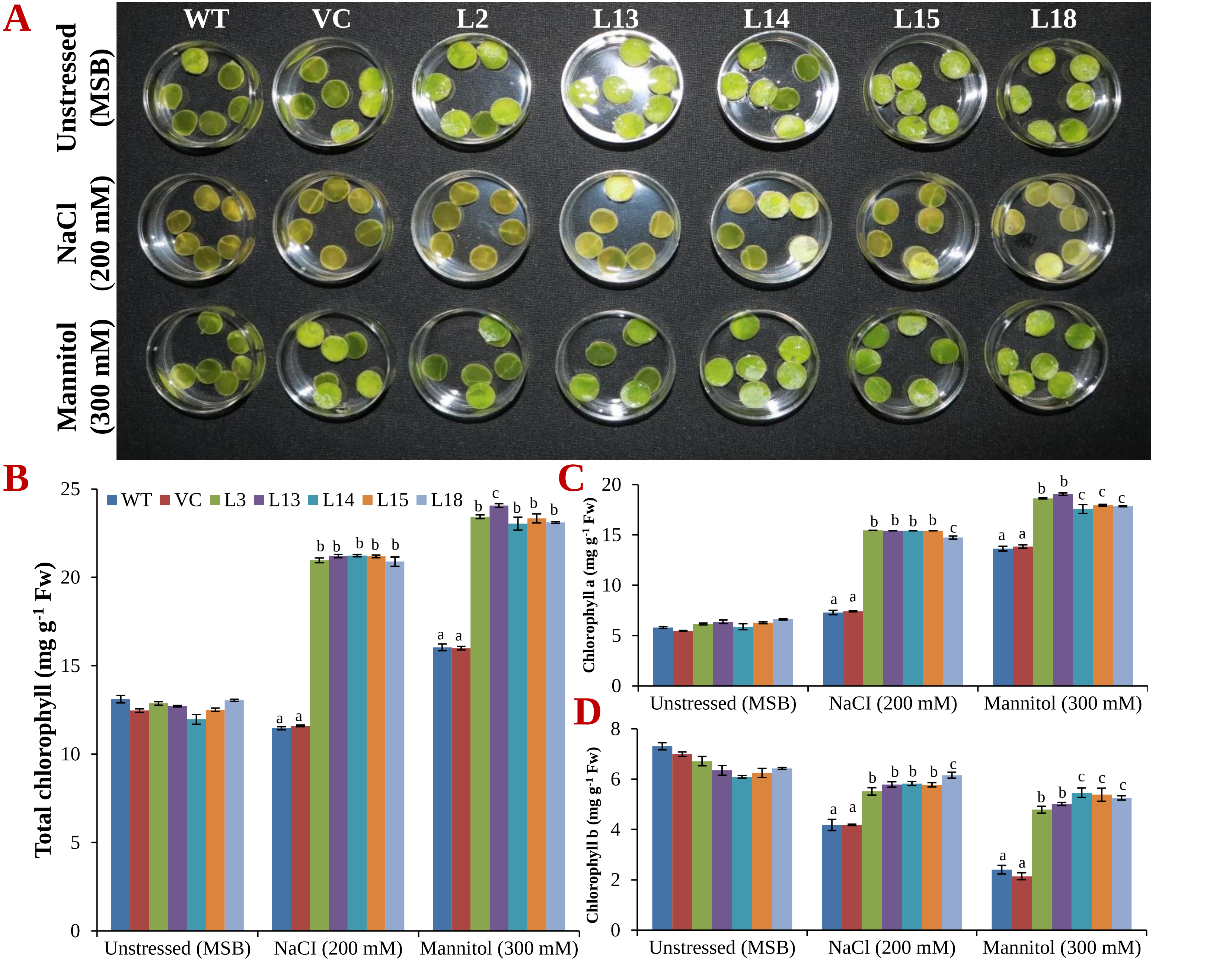

Figure S5: Leaf senescence and chlorophyll content analysis. The senescence assay **(A)** showed reduced chlorosis and less damage (necrosis) in transgenic compared to control plants and unstressed conditions. Further transgenic lines showed high **(B)** total chlorophyll, **(C)** chlorophyll a, and **(D)** chlorophyll b under salinity and osmotic stress conditions. Bars represent means ± SE and values with different letters are significant at $p < 0.05$.

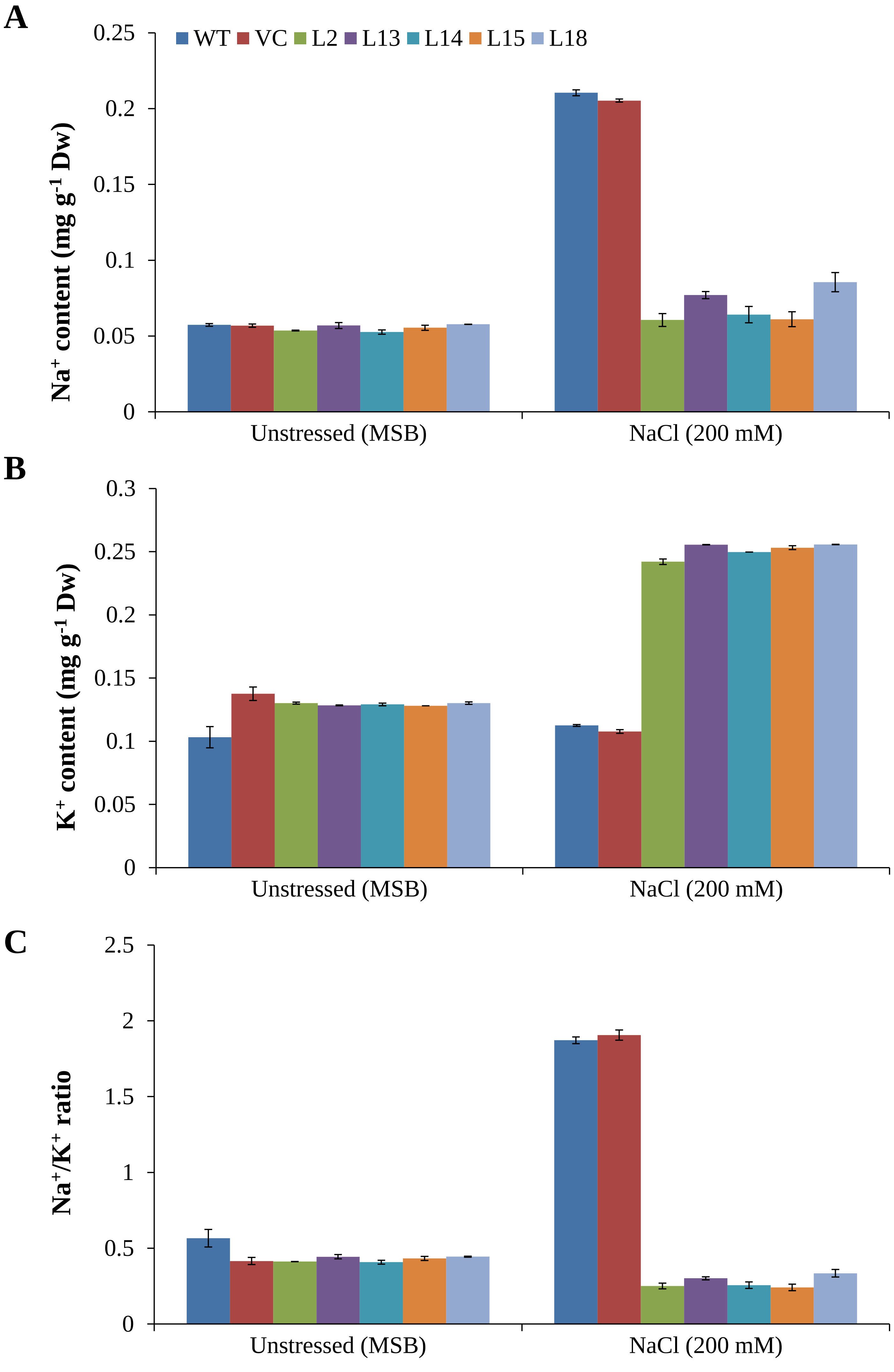

Figure S6: Ion content analysis. (A) Na ion, **(B)** K ion and **(C)** Na^+/K^+ ratio were estimated in the transgenic plants under salt stress condition and compared with control plants. Bars represent means ± SE and values.

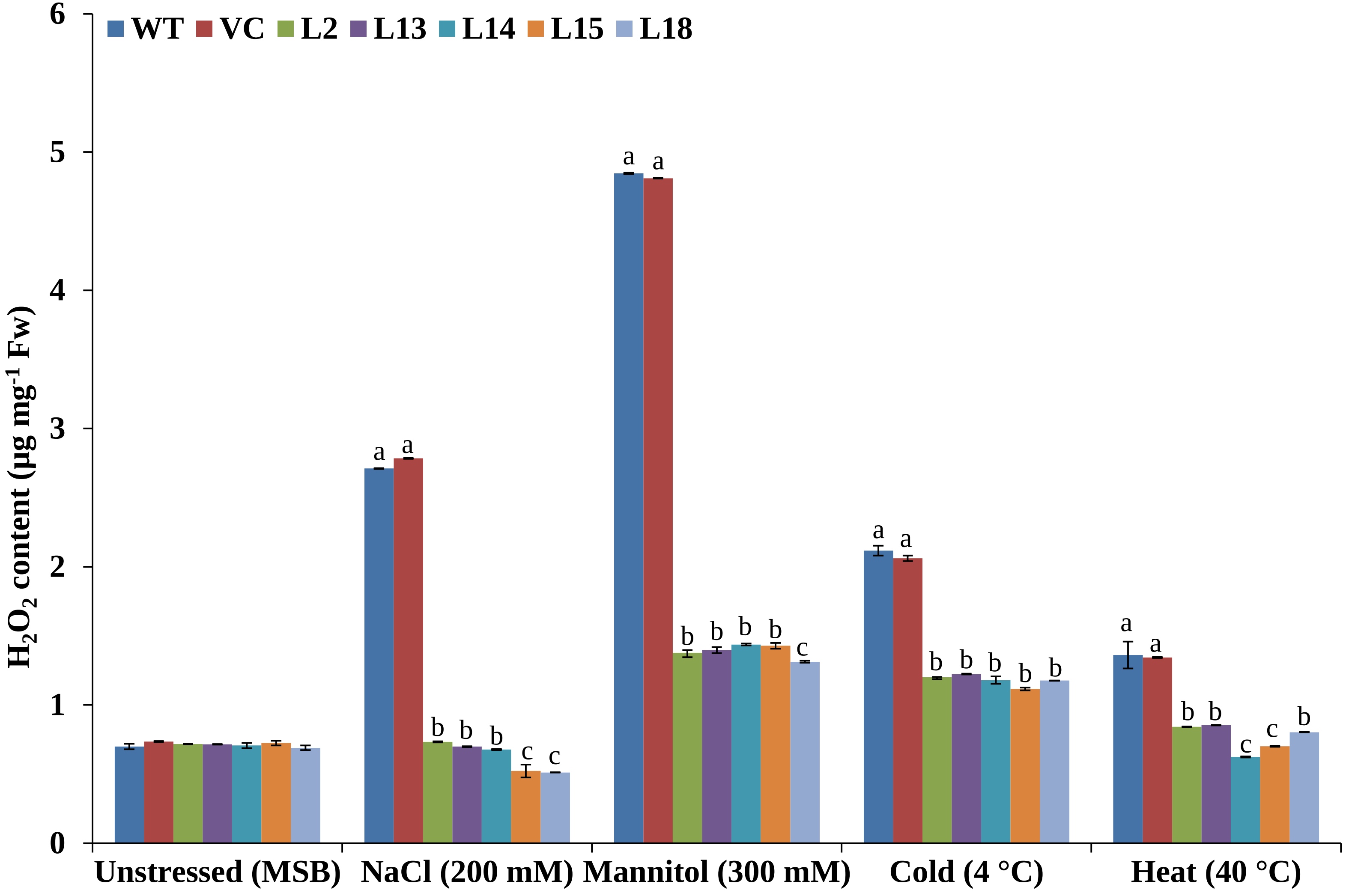

Figure S7: Analysis of H_2O_2 content. Quantitative analyses of H_2O_2 content of transgenic lines (L2, L13, L14, L15 and L18) and control plants (WT and CV) grown under unstressed (MSB), salt (200 mM NaCl), osmotic (300 mM manitol), cold (4 °C), and heat (40 °C) stress conditions. Bars represent means ± SE and values with different letters are significant at $p < 0.05$.

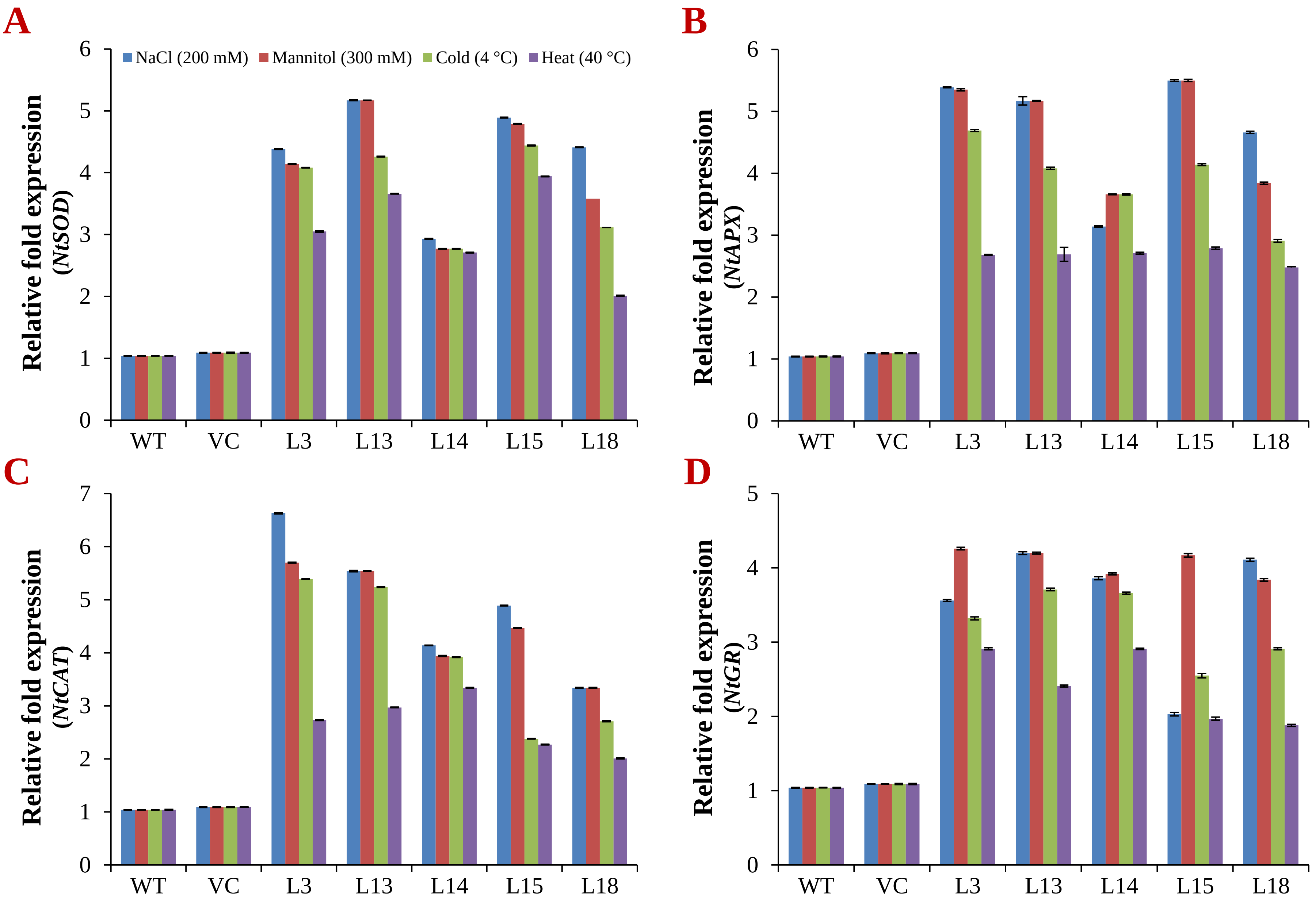

Figure S8: Transcript expression analysis of ROS scavenging antioxidant enzyme encoding genes. The relative fold expression of **(A)** *NtSOD* gene encoding superoxide dismutases, **(B)** *NtAPX* encoding ascorbate peroxidase, **(C)** *NtCAT* encoding catalase, and **(D)** *NtGR* encoding glutathione reductase enzyme was analyzed under different stress conditions. Bars represent means ± SE and values.
